# In vivo three-dimensional kinematics of the cervical spine during maximal active head rotation

**DOI:** 10.1371/journal.pone.0215357

**Published:** 2019-04-16

**Authors:** Jian Kang, Guangru Chen, Xu Zhai, Xijing He

**Affiliations:** 1 Fifth Department of Orthopedics, Baoji Chinese Medicine Hospital, Baoji, Shaanxi Province, China; 2 Second Department of Orthopedics, Second Affiliated Hospital of Xi'an Jiaotong University Medical School, Xi’an, Shaanxi Province, China; Northwestern University, UNITED STATES

## Abstract

**Objective:**

The aim of this study was to measure the movement of the cervical spine in healthy volunteers and patients with cervical spondylosis (CS) and describe the actual motion of the cervical spine using a three-dimensional (3D) CT reconstruction method. The results can enrich current biomechanical data of cervical spine and help to find the differences between the noted two groups.

**Materials and methods:**

20 healthy volunteers underwent CT examination ranging from the clivus of the occiput (Oc) to the top of first thoracic vertebrae (T1) in a neutral position with left or right maximal axial rotation, while 26 CS patients received the same CT scan procedures in the neutral position with left and right maximum rotation. Subsequently, the three-dimensional images of the occiput and every cervical vertebrae (C1-C7) were reconstructed using medical software. 3 virtual non-collinear markers were placed on the prominent structures of foramen magnum and every cervical vertebrae. Then, the 3D orthogonal spatial coordinates were defined with these anatomical markers to represent the orientation and position of every vertebra. Segmental relative motions were calculated using Cardan angles in the 3D spatial coordinates. Finally, the differences between the two groups were analyzed with statistical software SPSS.

**Results:**

The cervical spine exhibited complicated 3D movements, which could be adequately described using the three-dimensional CT reconstruction method. Reliability analysis of the 3D CT reconstruction method showed inter-rater ICC of 0.90–0.99 and intra-rater ICC of 0.91–0.98, suggesting very good consistency. Besides, the rotation at the upper cervical spine (Oc-C2) took up at least 60% of the total cervical rotation. The coupled lateral bending movement of the upper cervical spine was opposite to the major motion, while the movement of the lower cervical spine followed the same direction as that of the major motion. Oc to C5 segments were all coupled with the back-extension movement. The relative translations of all adjacent segments in each direction were minimal. CS patients showed a significant decrease in the movement of the C4-C5 segment compared with healthy volunteers.

**Conclusion:**

The motion of the cervical spine was complicated and three-dimensional. The CT reconstruction method employed here was good at describing such movement. The 3D CT reconstruction method exhibited high reproducibility when measuring cervical spine movement. CS patients and healthy volunteers showed significant differences in the movement of some segments.

## Introduction

With the advancement of technology and the transformation in lifestyle, the incidence of cervical spondylosis (CS) has progressively increased. As one of the major symptoms of CS, the reduction in the range of motion (ROM) of cervical spine seriously reduces the life quality of patients [1]. Accordingly, CS was considered as the second most stubborn disease in the early 21^st^ century. However, ROM measurement of cervical spine in CS patients can significantly improve the diagnosis and treatment of CS.

Some studies have shown that the two-dimensional images of the spine and the movement on the two-dimensional images can be accurately and automatically depicted [2, 3], but more studies suggested that the movement of cervical spine is three-dimensional (3D). During the cervical spine movement in one direction (principal/main motion), its motion in other directions (coupled motion) is also present [1, 4–7]. Several researchers [4–6] aimed to measure the ROM of cervical spine in human cadavers, whereas they could only obtain inaccurate data. This is because the specimens lacked muscles attachment and a physiological load.

Furthermore, cervical spine in the measurements above showed passive movement. In recent years, in vivo studies showed higher reproducibility when measuring cervical ROM. As two examples of new technology, MRI and CT 3D reconstruction have been broadly employed to fully achieve active in vivo measurements [7, 8]. Besides, the visualization of 3D images and 3D orthogonal spatial coordinates helps to describe the real 3D movement of cervical spine with greater accuracy [8–10]. For instance, Nagamoto, et al. achieved the accuracy of 0.24° to 0.43° for angles and 0.41mm to 0.52mm for translation in their study [7]. However, the spatial resolution of MRI 3D reconstruction is definitely weaker than that of CT 3D reconstruction in bone tissues. Besides, during MRI scanning, each position requires at least 5 min of measurement, several times longer compared with CT measurements. This brings more difficulties for subjects to accomplish maximal active head rotation. Thus, the CT 3D reconstruction method appears to outperform MRI. To gain a deeper insight into the movement of cervical spine, this study was designed to carry out a quantitative comparison between healthy adult volunteers and CS patients using a definition flash CT technology.

## Methods and materials

### Ethics approval and consent to participate

This study was approved by the Ethics committee of Second Affiliated Hospital of Xi'an Jiaotong University Medical School and the Medical Ethics committee of Baoji Chinese Medicine Hospital.

All subjects were recruited between May 2017 and May 2018, including 20 asymptomatic healthy volunteers (10 males, 10 females; with the average age of 24 years; age range in 22–26 years) and 26 CS patients (2 males, 24 females; average age, 52 years; range, 36–72 years). Asymptomatic healthy volunteers were recruited openly for the society, and patients with cervical spondylosis were selected from hospital patients. All subjects had satisfied the inclusive criteria here. Definition flash CT (photon CT, Dual Source CT) was the major equipment applied here. Because of the exposure to radiation, all subjects were informed in advance, and informed consent was obtained, which was examined by the Ethics Committee and satisfied ethical requirements. All the authors had access to information that could identify individual participants during or after data collection. Compared with conventional CT, definition flash CT is superior in scanning speed, scanning time, the amount of radiation as well as image resolution. However, to reduce the radiation exposure, only the movement of maximal head rotation was measured.

### CT parameters

The parameters of definition flash CT applied here included: Manufacturer, Siemens; Origin, Germany; Manufacturing date, 09/2013; Model No., 10430603; Serial No., 73805. All images were acquired by definition flash CT with the following settings: Scan mode, Dual Energy; voltage of A and B Bulb Tubes, 100KV and 140KV, respectively; Slice thickness, 0.625mm; Beam collimation, 0.6mm; Pitch, 0.7; Beam pitch, 0.9; Tube rotation speed, 0.5 second per circle.

### CT scanning and reconstruction

All subjects underwent definition flash CT scan from clivus to the upper edge of the first thoracic vertebral. In the control group (healthy volunteers), the images were captured at the neutral position and randomly assigned maximal left or right rotation to simulate the movement of cervical spine. In the experimental group (CS patients), the images at the noted positions were captured. During imaging process, all subjects laid supine on the CT working bench with 2 different planes of image acquisition perpendicular to the working bench. One plane was the Frankfurt horizontal plane (F-H plane), including both sides of the image portion and the obitale [9, 10], while the other plane was the sagittal plane of the subject [8]. During the scan, two belts were employed to tie the shoulder and chest of each subject to the CT working bench to eliminate the movement of the upper thoracic spine, which could potentially vary the cervical spine movement by up to 20° [11].

**Fig 1:**
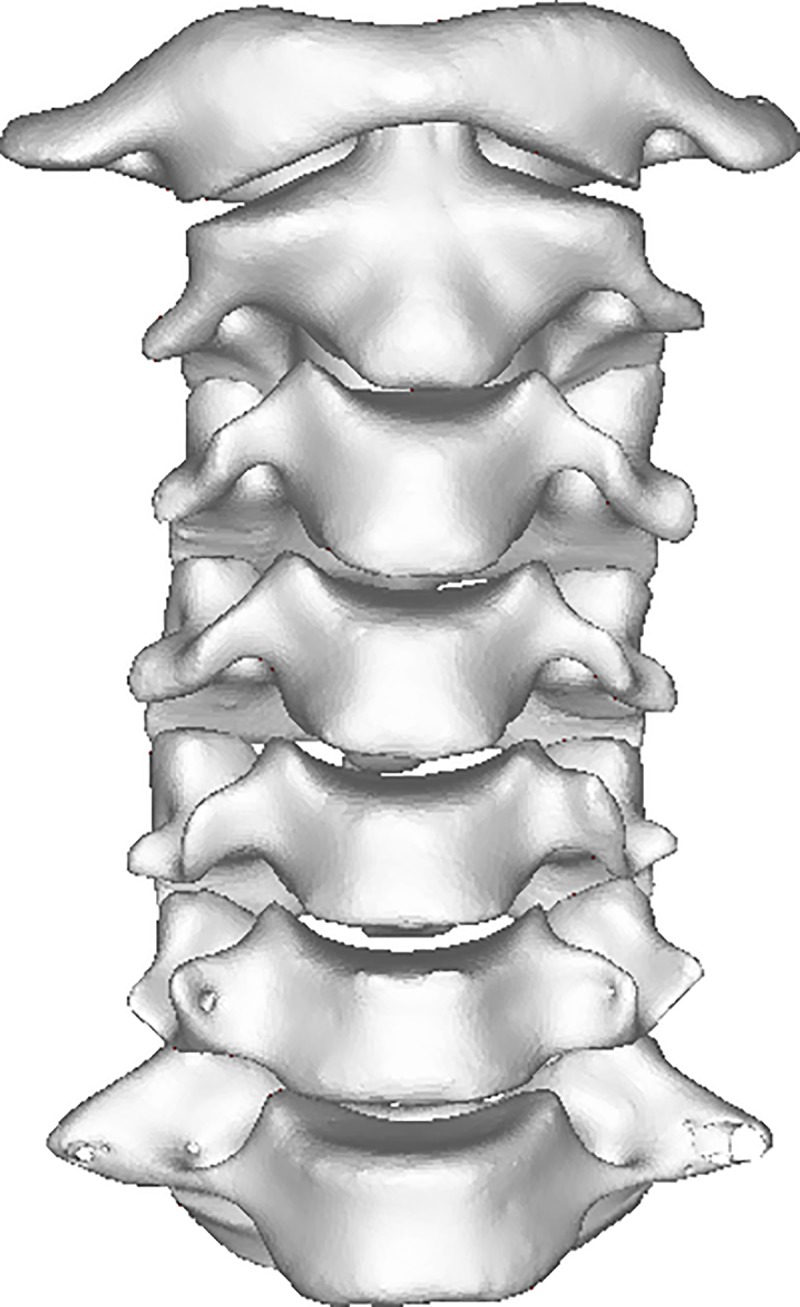
CT 3D reconstruction image of cervical spine: Anterior view.

**Fig 2:**
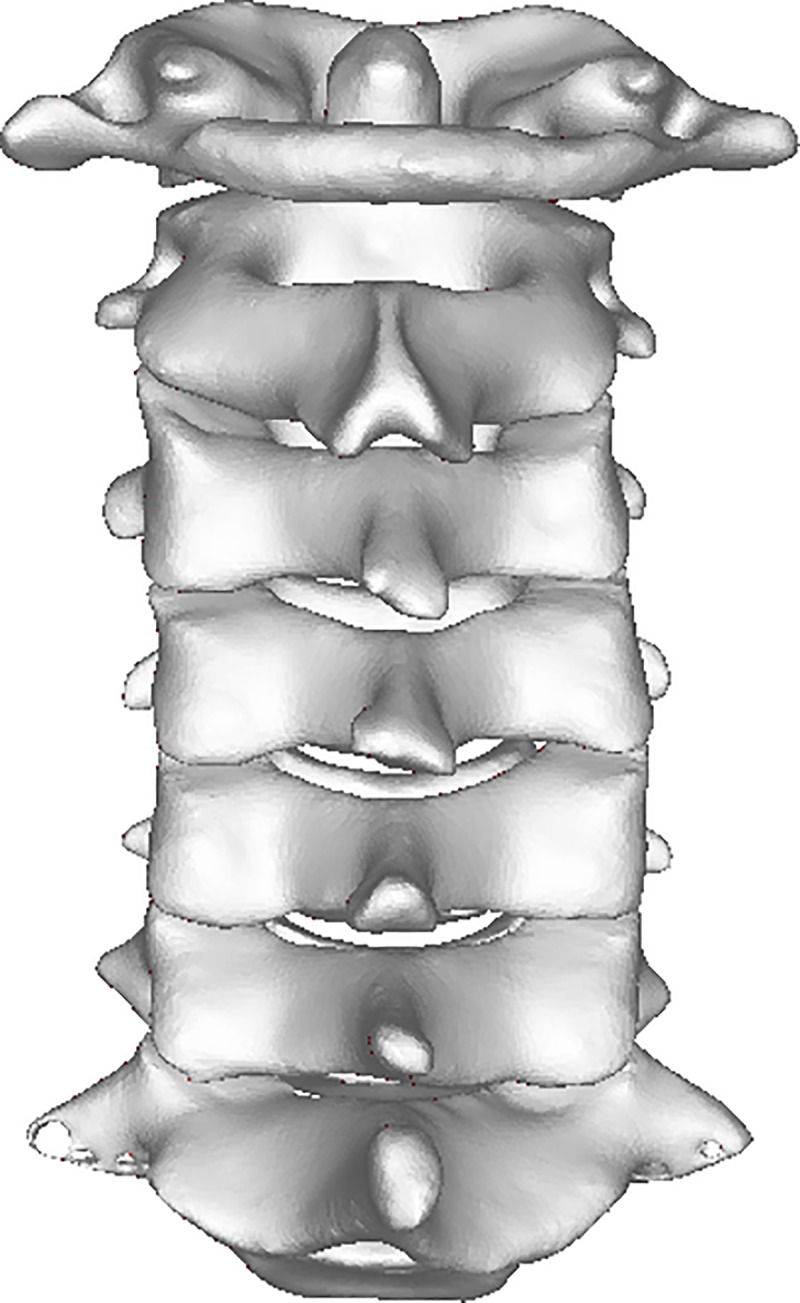
CT 3D reconstruction image of cervical spine: Posterior view.

The CT scanning data were acquired in DICOM format and then directly imported into medical software Mimics. By setting the threshold as “Bone (CT)” in the software, the images of bone tissues were extracted from original CT data. Subsequently, the images were segmented and filled to reconstruct each layer of the 3D images (Fig 1 and Fig 2).

### Data analysis

**Fig 3:**
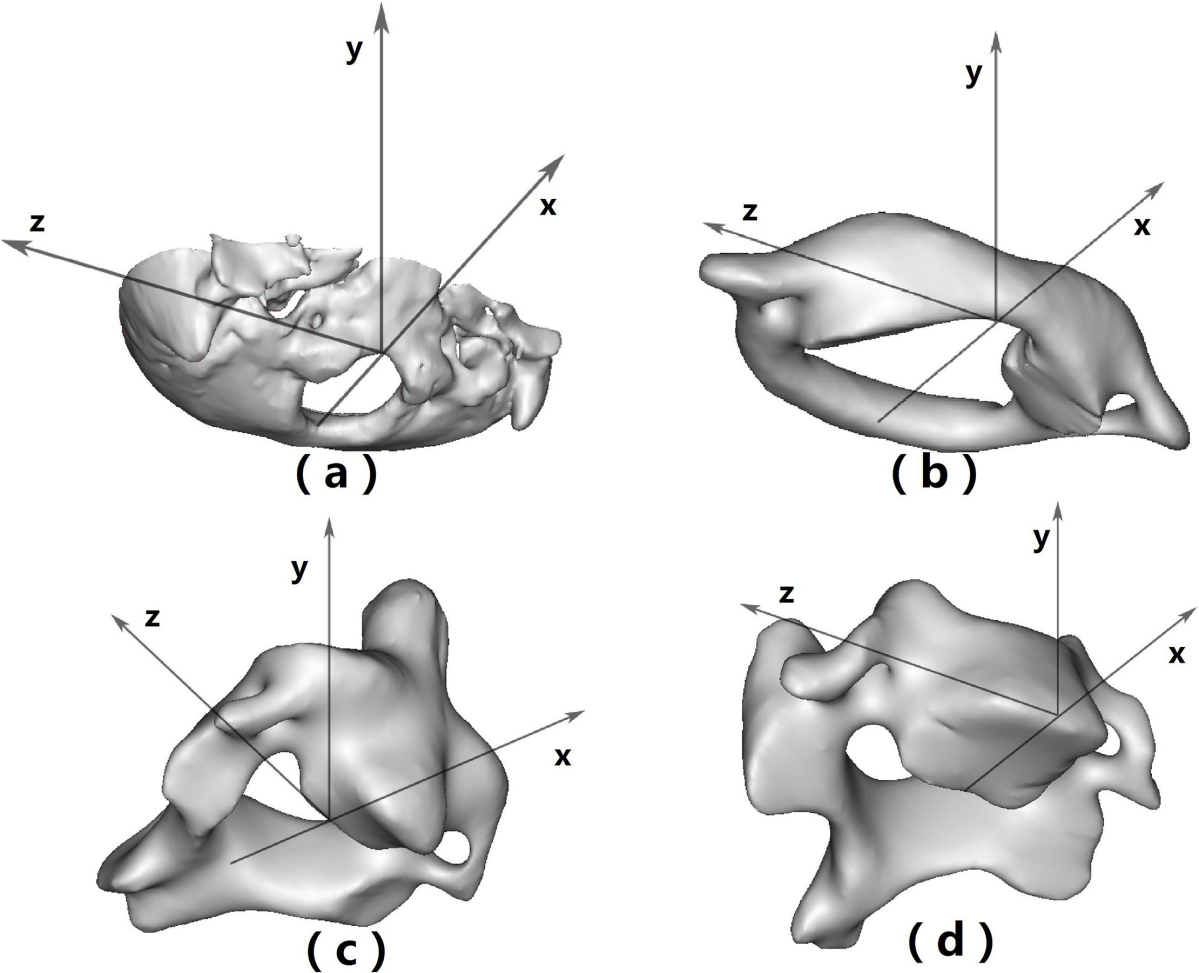
The LCS in different vertebrae (occiput): A Local Coordinate System (LCS) was established for Occiput (Oc) (a), the first cervical vertebrae (C1) (b), the second cervical vertebrae (C2) (c) and the lower cervical vertebrae (d).

The cervical spine movement was measured by calculating the variations in the position of each pair of adjacent vertebrae, while a Local Coordinate System (LCS) defined by the location of certain anatomic points and planes on each vertebral could be employed to completely represent the position of corresponding vertebrae (e.g., their location and orientation) in accordance with the theory of Rigid Body. The LCS (Fig 3) applied here employed the following configurations: (a) Occiput (Oc): the x-axis was defined as the line connecting two points on the anterior and posterior borders of foramen magnum on the sagittal plane, with the popup located at the anterior border deemed positive. For the two points, one was the most anterior-inferior point on the posterior border, while the other was the most posterior-inferior point on the anterior border, which was also considered as origin. The y-axis was the line orthogonal to the x-axis on the sagittal plane, with the superior direction, which was defined as the positive direction. The z-axis was perpendicular to the sagittal plane with the right direction, which was considered as the positive direction; (b) the first cervical vertebrae (C1): the same as Oc; (c) the second cervical vertebrae (C2): basically the same as Oc except the differences in selecting the following two points, namely one was the most posterior-inferior point located on the posterior wall of vertebral body in the sagittal plane, while the other was the most anterior-inferior point located on the lamina; (d) the lower cervical vertebrae: Similar to C2, with one point being the most posterior-inferior point on the posterior wall and the other point being the most anterior-inferior point on the anterior wall of vertebral body in the sagittal plane. Compared with LCS, a Global Coordinate System, the basic coordinate system in data processing, was also defined in Mimics. Cardan Angles were used to assess the angle change in 3D space, with its translations directly calculated using a matrix. Besides, all results were represented by 6 degrees of freedom, namely Axial Rotation (AR), Coupled Lateral Bending (Coupled LB), Coupled Flexion/Extension (Coupled F/E), Coupled Lateral Translation (LT), Coupled Anterior/Posterior Translation (APT) as well as Coupled Superior/Inferior Translation (SIT). The statistics analysis of the data was conducted by T-tests or Mann-Whitney U tests according to the results from the test of normality, and p< 0.05 expressed significant difference.

### Reliability

Since accidental and human errors may occur during the placement of anatomical landmarks, a reliability test was performed before the results were analyzed. During the reliability test, 4 typical or special segments (Oc-C1, C1-C2, C3-C4 and C4-C5) from 20 healthy subjects were selected and operated by two operators, namely A and B. Subsequently, the results were compared in the test.

## Results

### Reliability

The inter-class correlation coefficient (ICC) of all selected segments was 0.91–0.98, and the intra-class correlation coefficient (ICC) was 0.90–0.99. These results exhibited huge reliability during the placement of anatomical points.

### 3D kinematics

**Fig 4:**
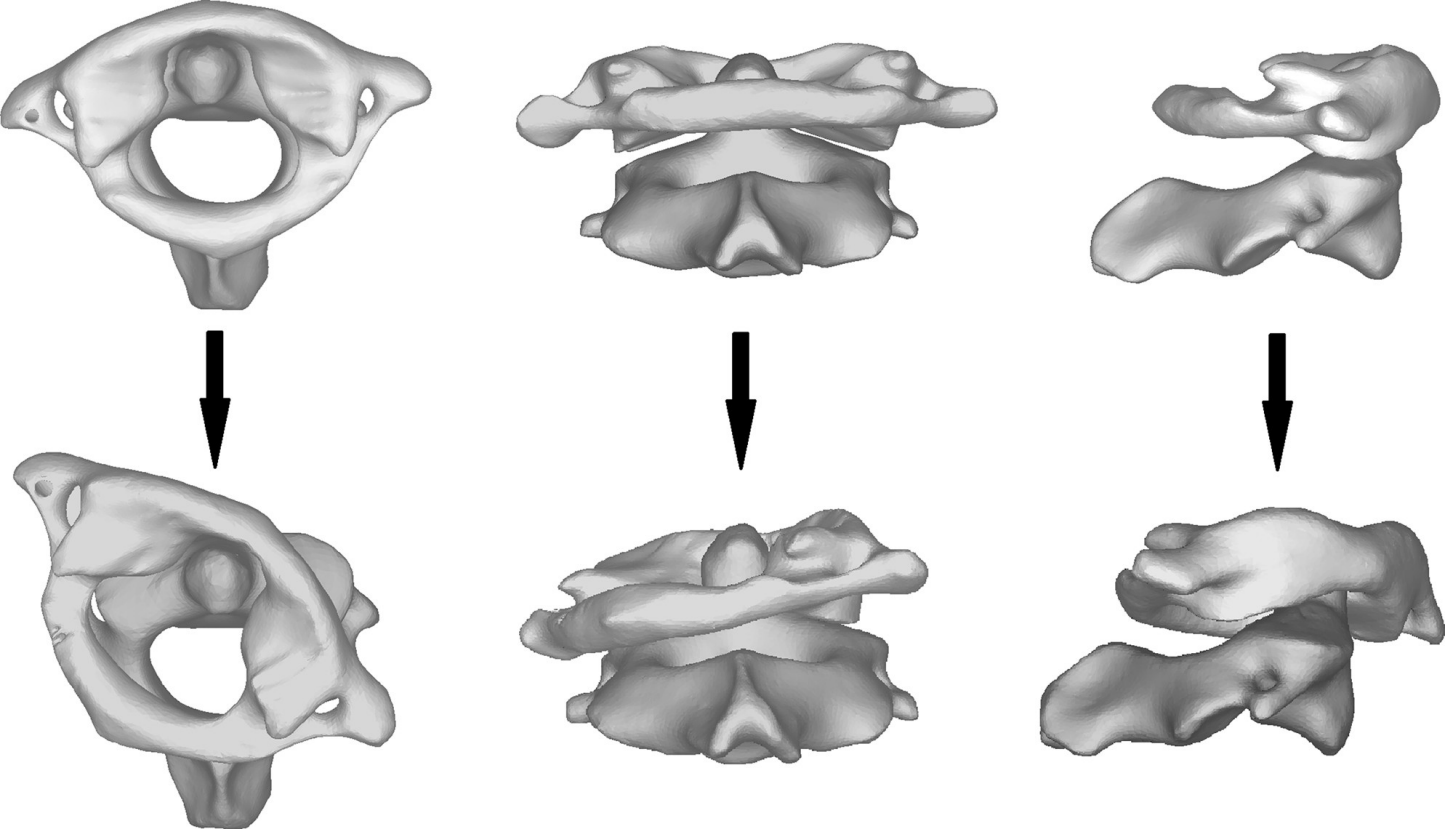
The movement of C1-C2 segment when the head rotated to the right side.

**Fig 5:**
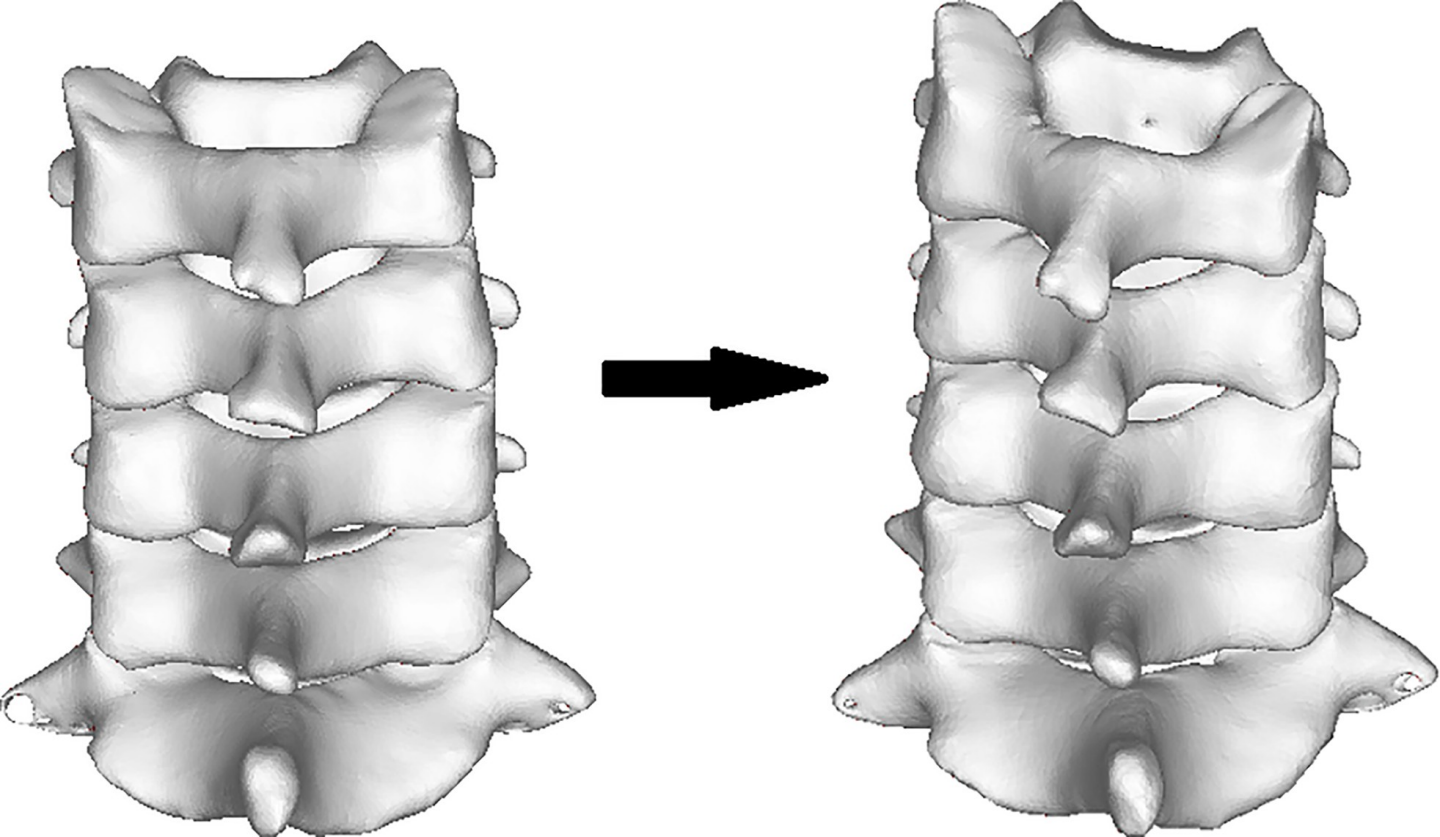
The movement of lower cervical spine when the head rotated to the right side.

The movement of cervical spine refers to a complicated 3D process involving some obvious translocation between the upper cervical spine (the UCS) and lower cervical spine (the LCS). When a head rotated to the right, the axial rotation was also pointed to the right, while the coupled lateral bending pointed to the left and a certain degree of coupled flexion occurred in the C1-C2 segment simultaneously (Fig 4). However, in the lower cervical spine, right lateral bending was coupled with right axial rotation (Fig 5).

### Axial rotation and coupled motion

**Fig 6:**
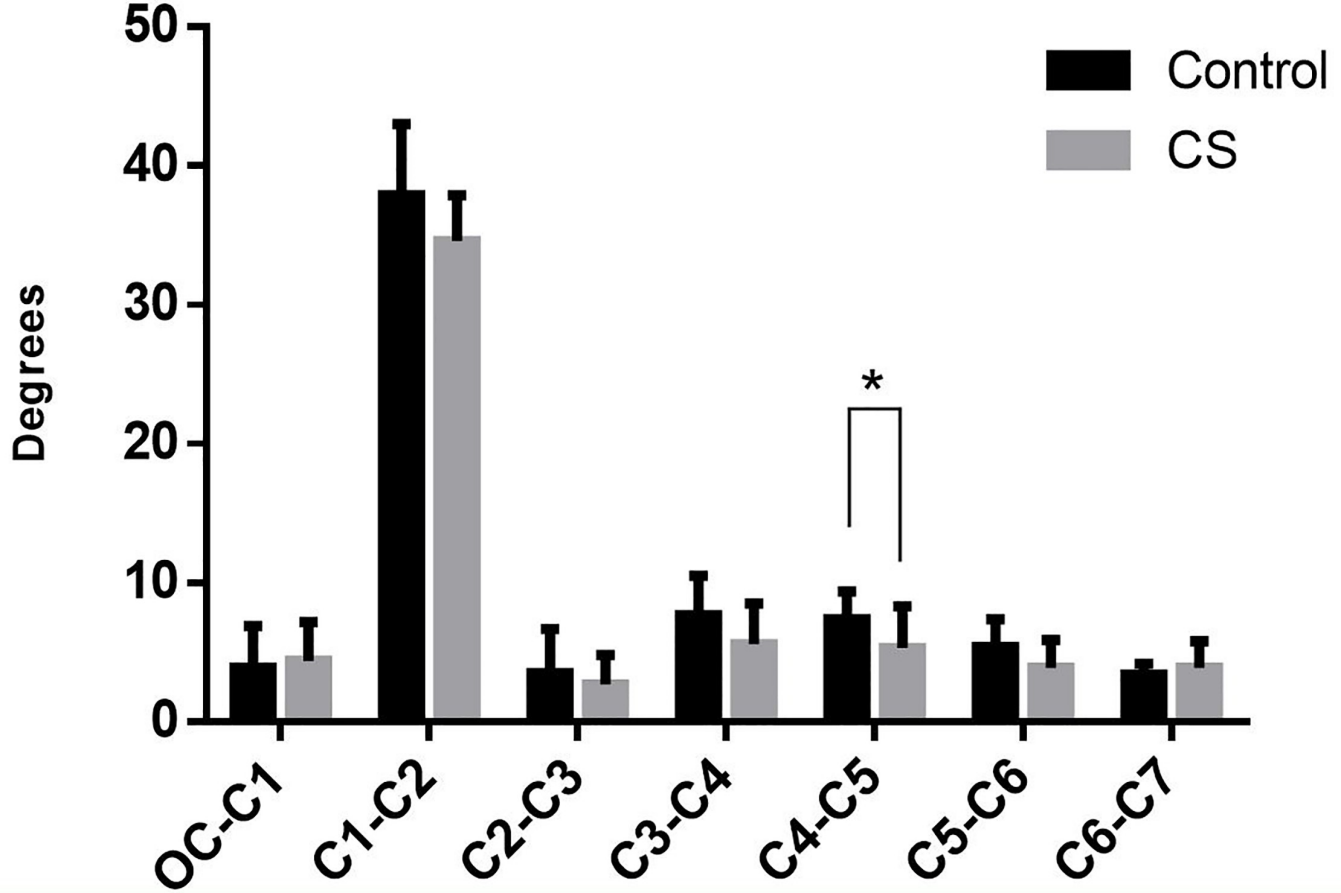
Axial rotation of each adjacent segment (positive values indicated the same direction of rotation): The data from the Control group and the CS patient group were compared. The difference at C4-5 was significant (P<0.05).

Compared with the healthy volunteers, a significant decrease (P<0.05) in the mean axial rotation was observed in CS patients when the head exhibited maximal active rotation at C4-5. Both two groups showed that the rotation at C1-C2 segment was proportional to total axial rotation (Fig 6).

**Fig 7:**
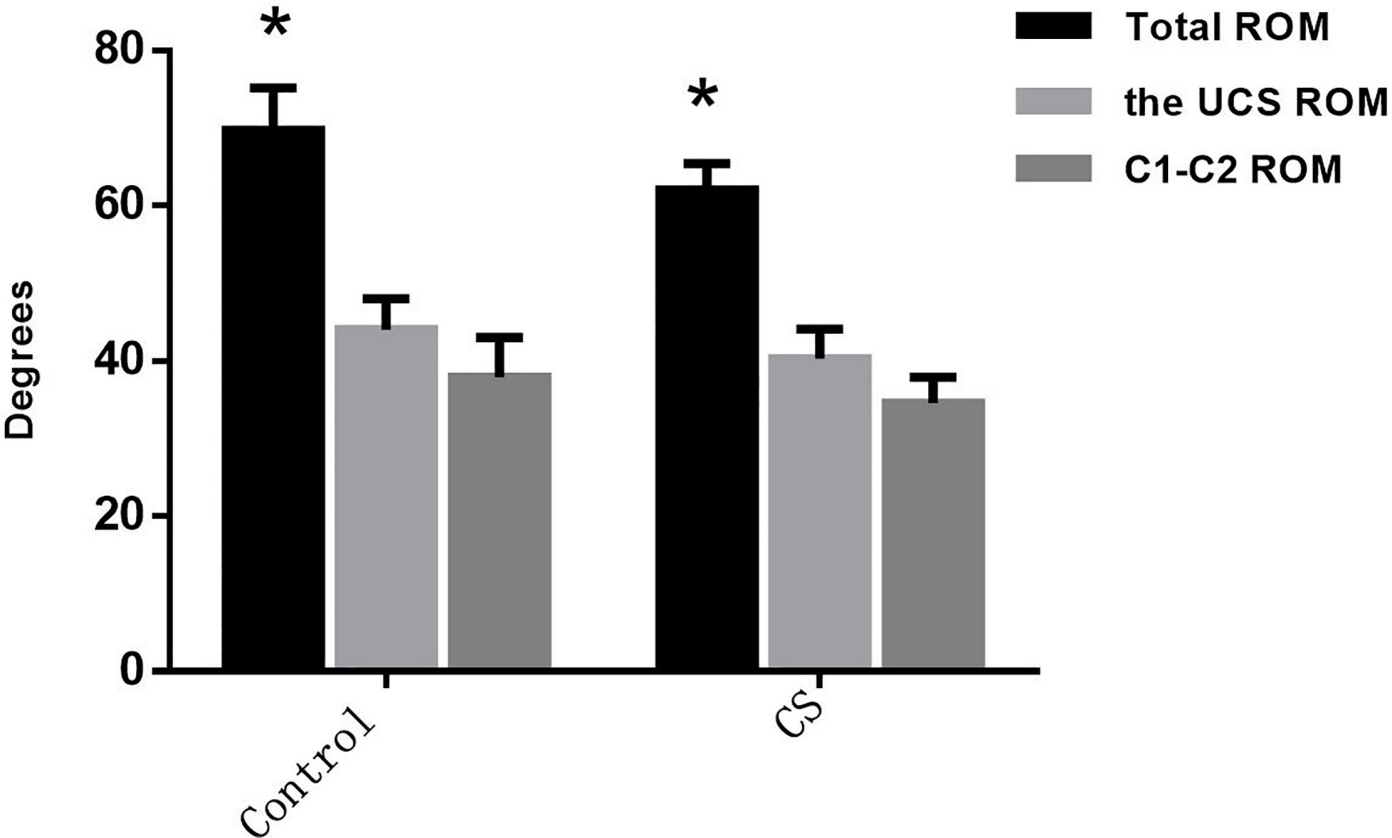
Rotations in different parts of the cervical spine: The upper cervical spine (UCS) contained two segments, Oc-C1 and C1-2. The difference of the entire cervical spine was significant (P<0.05).

In the control group, the rotation at different parts of the cervical spine was (69.7±5.5)° for the total rotation of cervical spine; (44.0±8.0)° for the upper cervical spine (UCS), which took up 63.13% of total rotation; (37.9±5.1)° for C1-C2 segment, taking up 86.14% of the rotation of upper cervical spine and 54.40% of the total rotation of cervical spine, respectively. In the CS group, the rotation was (62.0±3.4)° for the total rotation of cervical spine; (40.3±3.8)° for the UCS, taking up 65.00% of total rotation; (34.6±3.3)° for C1-C2 segment, accounting for 85.90% of the rotation of upper cervical spine and 55.80% of the total rotation of cervical spine, respectively. Compared with healthy volunteers, CS patients showed both decreased rotation in different parts of the cervical spine and decreased total rotation of cervical spine (P<0.05). (Fig 7)

**Fig 8:**
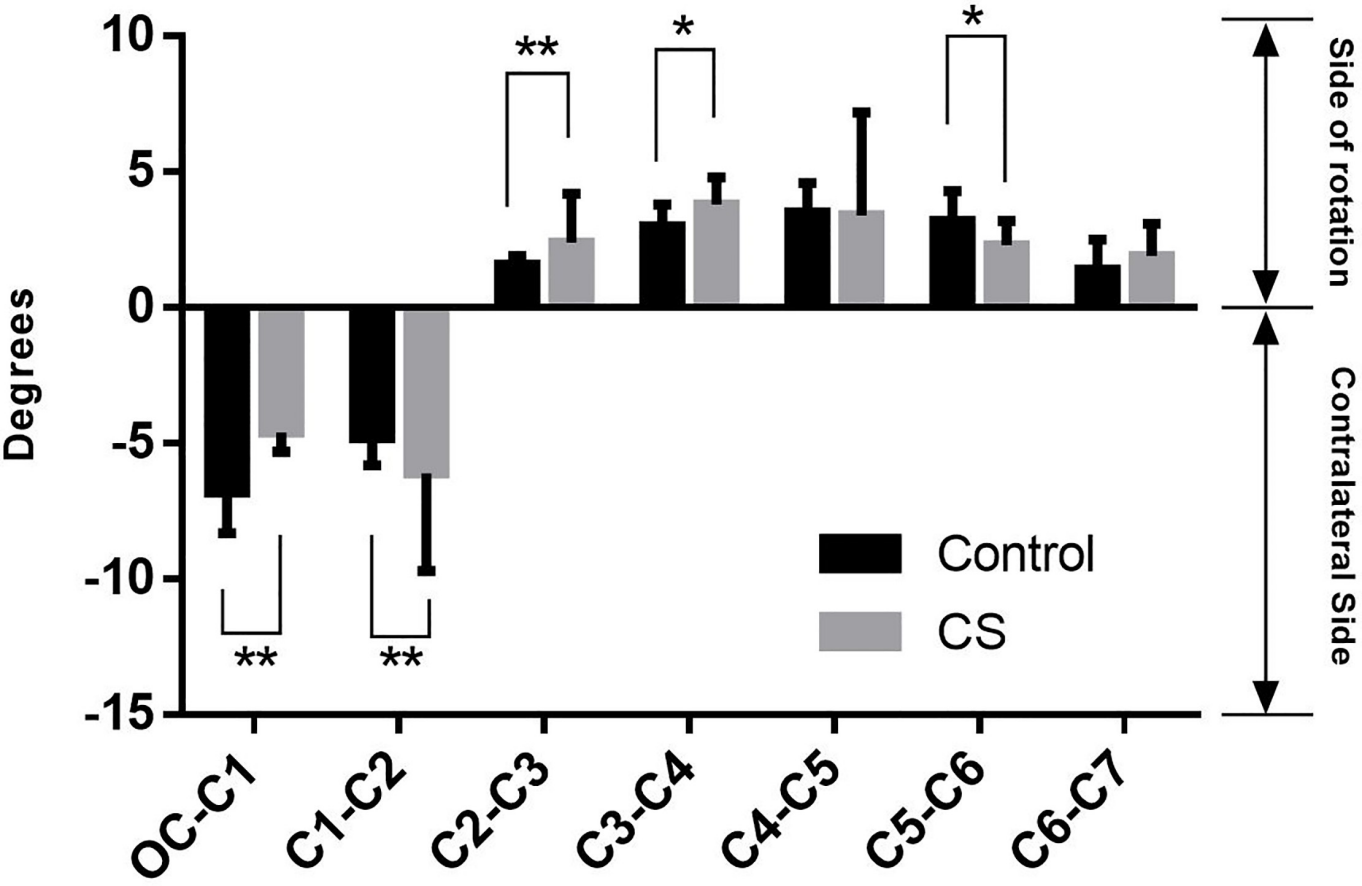
The lateral bending movement coupled with head rotation: Positive (negative) values represented the same (opposite) direction as that of head rotation.

When a head rotated to its maximum displacement, the lateral bending at the same side of lower cervical spine and at the contralateral side of the upper cervical spine rotation was all coupled with axial rotation in both groups. Significant decrease at Oc-C1 (P<0.01) and C5-C6 (P<0.05), as well as significant increase at C1-C2 (P<0.01), C2-C3 (P<0.01) and C3-C4 (P<0.05), were also found in the CS patients (Fig 8).

**Fig 9:**
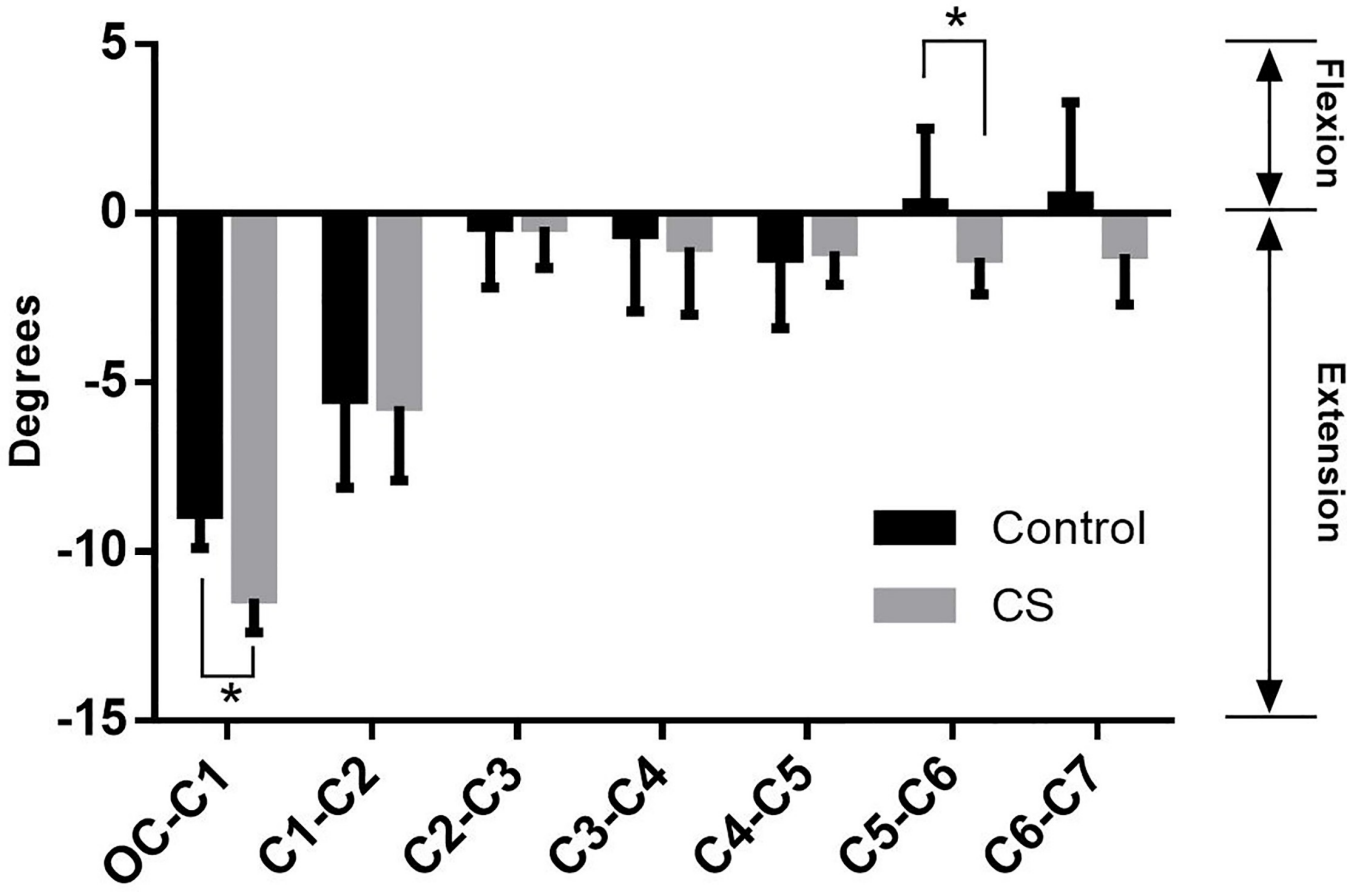
The flexion/extension movement coupled with head rotation: Positive (negative) values represented the flexion (extension) movement.

In both groups, coupled extension movement was observed in Oc-C1 to C4-C5 segments, while potential flexion movement was observed in C5-C6 to C6-C7 segments. Furthermore, significant increase was found in the Oc-C1 segment of CS patients (P<0.05) (Fig 9).

**Fig 10:**
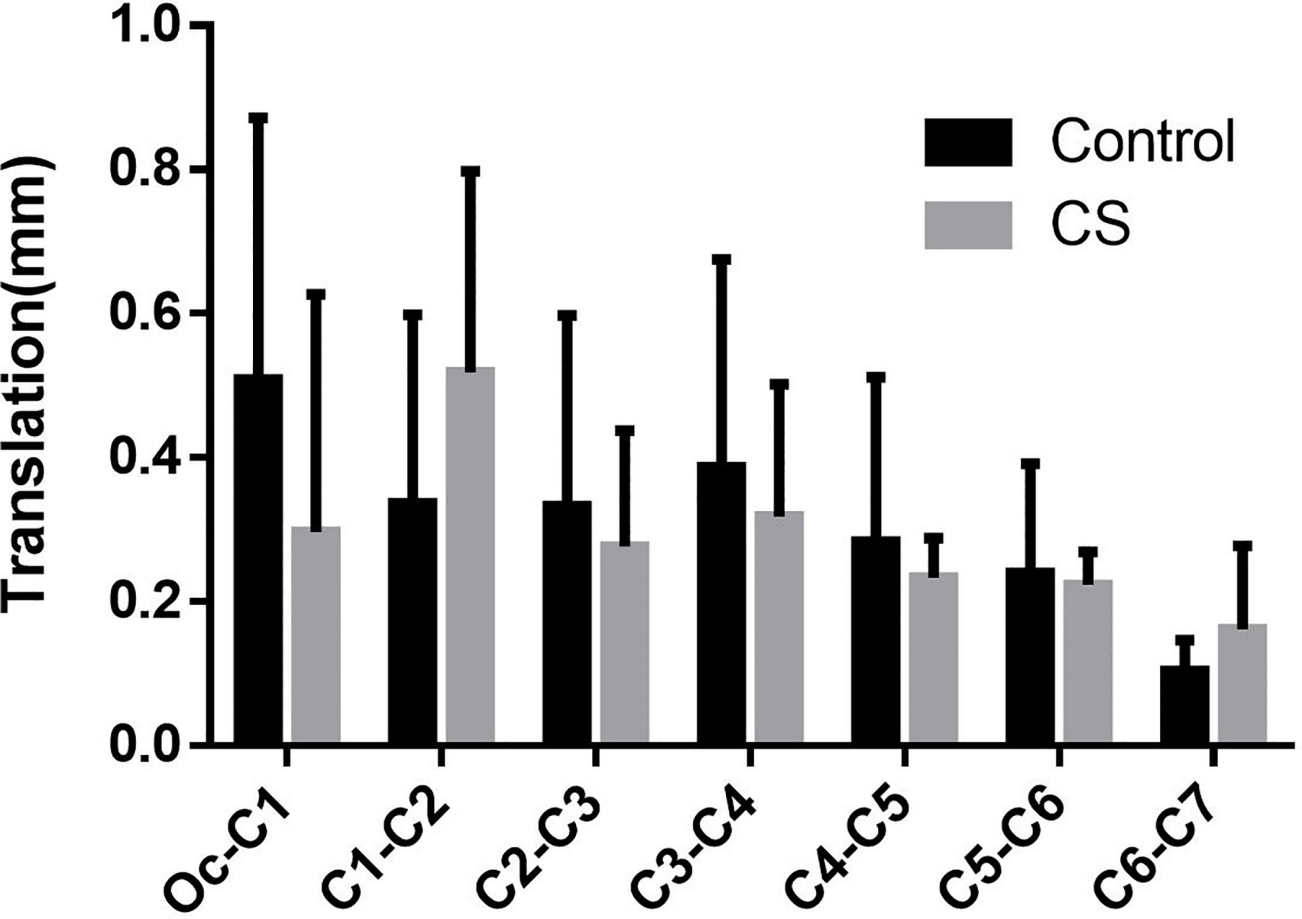
The lateral translation coupled with head rotation.

**Fig 11:**
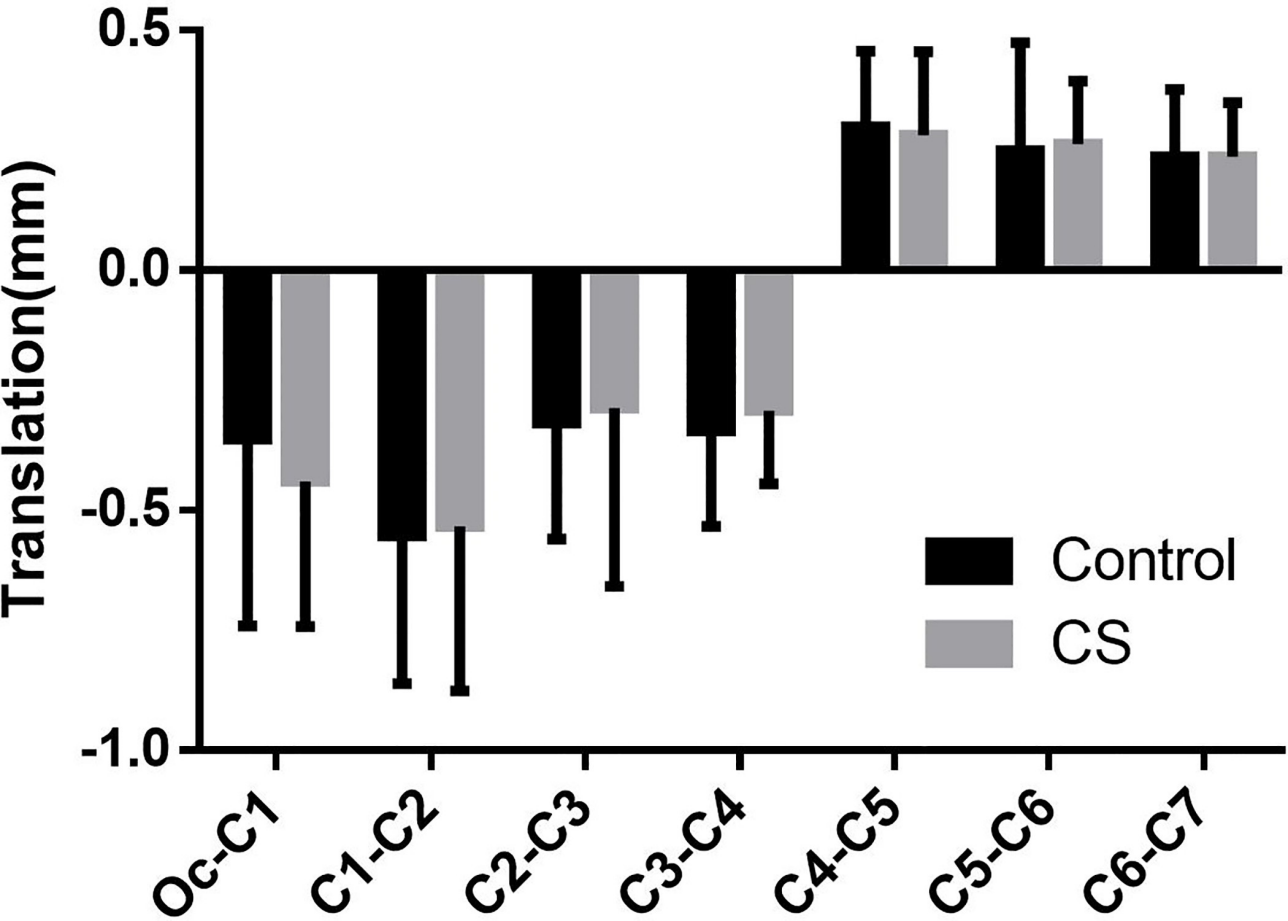
The anterior-posterior translation coupled with head rotation.

**Fig 12:**
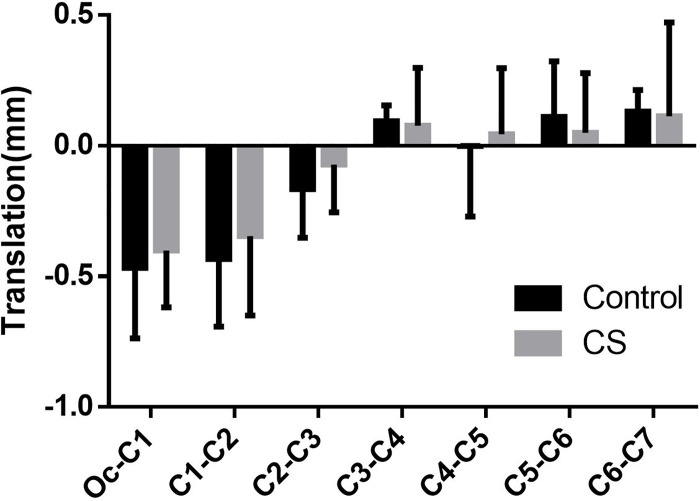
The superior-inferior translation coupled with head rotation.

Besides the coupled rotation movement, translations in the direction of axes also occurred simultaneously. Nevertheless, the results here revealed that the translations in all directions were small and insignificant between the two groups (Figs 10–12).

## Discussion

In this study, qualitative and quantitative measurements for the ROM of cervical spine in both CS patients and healthy volunteers were performed using CT 3D reconstruction, the most reliable and efficient method currently available. The movement of cervical spine refers to a complicated compound motion. When head rotates to one side, its axial rotation is called principal motion, while the other movements of the head (e.g., lateral bending, flexion or extension and translations) are overall named coupled motion.

## Axial rotation

### The upper cervical spine

The ROM at Oc-C1 segment has been rarely reported in previous studies on cadavers, though Panjabi.et al. [12] suggested a 7.3° maximum rotation of the head in their in-vitro study. During other in vivo measurements, the ROMs at Oc-C1 segment were measured in healthy subjects using different methods, yet the results were nearly the same: 1.7° measured by a MRI 3D reconstruction method [13], and (2.4±1.8)° and (2.5±1.0)° measured by a CT 3D reconstruction method [1, 8]. In addition, the ROMs followed the same direction as that of head rotation. However, ROMs with opposite direction as that of head rotation were also observed with Biplane X-Ray [14]. In the present study, (3.9±3.0)° and (4.4±2.8)° were obtained in the control and CS groups, respectively, and the direction was the same as that of head rotation.

Compared with the ROM at C1-C2, i.e., (37.9±5.1)° and (34.6±3.3)° for the control and CS groups, respectively, the ROM at Oc-C1 was 5 times higher than the degree of rotation and their special anatomical structures may explain such results.

Bogduk, et al. [15] considered flexion or extension movement as the only major motion at Oc-C1, and because of the limitation in the cradle structure of atlanto-occipital joint and the lack of direct muscle involvement in the rotation, the movement of rotation was always the passive and coupled motion. In contrast, the disappearance of intervertebral disc and convex articular surface on the lateral mass, as well as the appearance of odontoid and the formation of atlantoaxial joint with C1, all contributed to the larger rotation of C1-C2 [8]. Besides the articular capsule, alar ligament was also considered as the major factor limiting the rotation of upper cervical spine [15, 16], though individual differences still exist [8, 17]. Thus, the atlanto-occipital joint works for flexion-extension movement, while atlantoaxial joint contributes to rotation [18].

#### The lower cervical spine

The rotation of C2-C3 segment was the smallest among all segments in the lower cervical spine. Bogduk, et al. [15] attributed it to the orientation of the articular surface of zygapophysial joints. Due to the instability of rotator muscles after trauma and ligament damage, the rotation was found to occur in a totally opposite direction [19].

The C3-C4 and C4-C5 segments exhibited relative large rotation, which may serve as the main factor to maintain physiological head rotation. In the lower cervical spine, the rotation of C2-C3 was limited by its special anatomical structures. Besides, the C5-C6 and C6-C7 segments were also confined by the connective thoracic vertebrae, thereby exhibiting the smallest ROM in the entire spine. Accordingly, to complete the head rotation, the ROM of C3-C4 and C4-C5 segments had to increase in a compensatory fashion.

#### Comparison

The average ROM in the rotation of C4-C5 segment in CS patients significantly decreased compared with that of the healthy subjects, which may be attributed to special biomechanical factors and relative large ROM of the C4-C5 segment. As one of the most obvious lordosis segment, C4-C5 bore the major pressure from head, which could promote the degeneration of this segment. Furthermore, the above observations could also be explained by the number of patients (20/26) with lesions in the C4-C5 segment. Besides, the ROM of each lower cervical spine segment decreased to a certain extent, which was probably caused by age related degeneration to the muscles of the neck [20].

### The coupled motion

#### Coupled flexion/extension and lateral bending

In the upper cervical spine, the lateral bending and extension in the contrary side of rotation were coupled [8, 13], though some researchers [15] believed that the lateral bending and extension depended on the strength of head rotation and the shape of atlantoaxial joint regardless of whether flexion or extension is coupled with axial rotation. In addition, the alar ligament is still thought as the main factor responsible for the later bending at the contrary side.

In the lower cervical spine, the lateral bending in the same side was also coupled [1, 8, 21], which may be correlated with the direction of zygapophysial joints. [21] Compared with the healthy subjects here and of other studies [8, 21], the movement of C6-C7 segment in the sagittal plane of CS patients was extension, which may be attributed to a higher average age and the presence of kyphosis and stiffness [8] in CS patients.

#### Coupled translation

The coupled translation in each direction was small and insignificant between the two groups, which is consistent with the results from a previous study [7]. As expected, the translations were limited to keep the balance between stability and motility of the cervical spine.

#### Mechanism

Though the exact mechanism of coupled motion remains elusive, it may cover the following factors: the uncinate process orientation of C2-C7 [22]; the deformation of soft tissues under pressure [23]; the effect of the main head movement on the orientation of spiral axis [24]; the inclination of articular surface from C2 to C7 [25]; the curvature of the cervical spine, and the gravity [8].

## Conclusion

The movement of cervical spine is complicated and compound, and the CT 3D reconstruction is a reliable method capable of accurately measuring the ROM of cervical spine. However, a small sample size was the major limitation of this study. Besides, the supine CT scanning position could not describe the real movement of cervical spine under a physiological load. Thus, further studies are required to solve such problems.

## Supporting information

S1 File STROBE_checklist_v4_combined_PlosMedicine. (DOCX)

## Abbreviations

3D: Three-dimensional
CS: Cervical spondylosis
ROM: Range of motion
UCS: Upper cervical spine
AR: Axial Rotation
LB: Lateral Bending
F/E: Coupled Flexion/Extension
LT: Lateral Translation
APT: Anterior/Posterior Translation
SIT: Superior/Inferior Translation
LCS: Local Coordinate System
